# Exploring primary healthcare practitioners’ experiences regarding the coronavirus disease 2019 (COVID-19) pandemic in KwaZulu-Natal, South Africa

**DOI:** 10.1017/S1463423622000536

**Published:** 2022-11-04

**Authors:** Gugu Gladness Mchunu, Orlando Harris, Celenkosini Thembelenkosini Nxumalo

**Affiliations:** 1 Faculty of Health Sciences, Durban University of Technology, Durban, South Africa; 2 Department of Community Health Systems, School of Nursing, University of California San Francisco, San Fransisco, CA, USA; 3 College of Health Sciences, School of Nursing and Public Health, University of KwaZulu-Natal, Durban, South Africa

**Keywords:** COVID-19, experiences, healthcare workers, pandemic, primary healthcare

## Abstract

**Background:**

The coronavirus disease 2019 (COVID-19) has spread rapidly around the world since the initial outbreak in Wuhan, China. With the emergence of the Omicron variant, South Africa is presently the epicentre of the COVID-19 pandemic in sub-Saharan Africa. Healthcare workers have been at the forefront of the pandemic in terms of screening, early detection and clinical management of suspected and confirmed COVID-19 cases. Since the beginning of the outbreak, little has been reported on how healthcare workers have experienced the COVID-19 pandemic in South Africa, particularly within a low-income, rural primary care context.

**Methods:**

The purpose of the present qualitative study design was to explore primary healthcare practitioners’ experiences regarding the COVID-19 pandemic at two selected primary healthcare facilities within a low-income rural context in KwaZulu-Natal, South Africa. Data were collected from a purposive sample of 15 participants, which consisted of nurses, physiotherapists, pharmacists, community caregivers, social workers and clinical associates. The participants were both men and women who were all above the age of 20. Data were collected through individual, in-depth face-to-face interviews using a semi-structured interview guide. Audio recordings were transcribed verbatim. Data were analysed manually by thematic analysis following Tech’s steps of data analysis.

**Results:**

Participants reported personal, occupational and community-related experiences related to the COVID-19 pandemic in South Africa. Personal experiences of COVID-19 yielded superordinate themes of psychological distress, self-stigma, disruption of the social norm, Epiphany and conflict of interest. Occupational experiences yielded superordinate themes of staff infections, COVID-19-related courtesy stigma, resource constraints and poor dissemination of information. Community-related experiences were related to struggles with societal issues, clinician-patient relations and COVID-19 mismanagement of patients.

**Conclusion:**

The findings of this study suggest that primary healthcare practitioners’ experiences around COVID-19 are attributed to the catastrophic effects of the COVID-19 pandemic with the multitude of psychosocial consequences forming the essence of these experiences. Ensuring availability of reliable sources of information regarding the pandemic as well as psychosocial support could be valuable in helping healthcare workers cope with living and working during the pandemic.

## Introduction

Coronavirus disease (COVID-19) is a type of severe pneumonia-like illness caused by severe acute respiratory syndrome coronavirus 2 (SARS-CoV-2) virus (Ma *et al.*, 2020). COVID-19 has spread rapidly around the world following an initial outbreak in Wuhan, China, and on the 30 January 2020 was declared a Public Health Emergency of International concern (Song and Karako, 2020). In 2020, COVID-19 was subsequently declared a pandemic owing to the rapid rise in global incidence rate and associated mortality (Ali *et al.*, 2020b). Since the occurrence of the first outbreak, there have been over 253 million confirmed COVID-19 cases and over 5 million deaths worldwide (World Health Organization, 2022). South Africa is presently the epicentre of the COVID-19 pandemic in sub-Saharan Africa (World Health Organization, 2022). As of 3 June 2022, the National Institute for Communicable Diseases (2022) recorded 3 965 422 cases with a 9.8% positivity rate. KwaZulu-Natal is among the top three leading regions in terms of COVID-19-related incidence, prevalence, morbidity and mortality in South Africa (Department of Health, 2021 and National Institute for Communicable Diseases (NICD, 2022). NICD (2022) reported that as of 3 June 2022, Gauteng had the highest proportion of new cases 30%, followed by the Western Cape 25 %, while KwaZulu-Natal accounted for 13%, Eastern Cape accounted for 11%, Free State, Mpumalanga and Northern Cape each accounted for 5%, North West accounted for 4% and Limpopo accounted for 2% of today’s new cases.

Globally, the number of infections and deaths continues to escalate with different waves of the pandemic occurring almost worldwide. Since the beginning of the outbreak, governments have instituted several measures to reduce the spread of infection and curb the multitude of effects that the COVID-19 pandemic has had on individuals, families, communities and society (Khanna *et al.*, 2020). There is evidence to suggest that the pandemic has compromised quality of life by increasing morbidity and reducing life expectancy (Vasishtha *et al.*, 2021). Furthermore, the economies of several nations, specifically South Africa and other African nations on the continent, have been negatively affected as the pandemic prolongs (McNeely *et al*., 2020; Youssef *et al*., 2021).

Recent epidemiological data on COVID-19 have revealed COVID-19 variants of concern (Alpha, Beta, Gamma and Delta) and variants of interest (Lambda and Mu) which pose a further threat to the health and well-being of individuals due to their inherent nature of heightened virulence and pathogenicity (WHO, 2021). The recent rapid rise in the number of new COVID-19 infections in South Africa prompted genomic sequencing which resulted in the detection of a new variant of COVID-19, the B1.1.529 clade which was reported on the 25 November 2021 (NICD, 2021). This new COVID-19 variant has subsequently been added to the existing cluster of COVID-19 variants of concern and was named the Omicron variant. Recent developments in the discovery and rollout of COVID-19 vaccines represent a curative approach to managing and preventing the disease burden related to infection (Coltart and Collet-Fenson, 2021). The combination of compliance to stipulated COVID-19 behaviour modification strategies and the successful scale-up of vaccine rollout provides hope for the improvement of the present predicament resulting from ravages of the pandemic.

Since the beginning of the outbreak, healthcare workers have been at the forefront in terms of preventive, promotive, curative and rehabilitative measures to manage the pandemic (Brophy *et al.*, 2021; Lewis and Mulla, 2021). In South Africa, healthcare worker, particularly at primary care level, has not only been involved in terms of clinical care but also successful implementation of the National Department of Health COVID-19 response strategy. With the continued upward trajectory of infection rates since March 2020, South Africa has experienced an unprecedented number of healthcare workers being infected by COVID-19 (Nxumalo and Mchunu, 2021). While data on the actual number of healthcare workers infected with COVID-19 remains inconclusive in South Africa, it is estimated that more than 1200 health workers have died from COVID-19 since the beginning of the outbreak (NICD, 2021). National estimates of infection rates in South Africa further suggest that the KwaZulu-Natal province is among the four leading regions with high incidence and mortality resulting from COVID-19 infection (Health, 2021). The inherent vulnerability to infection together with challenges in resources and infrastructure are some of the factors thought to contribute to the high rates of infection and deaths among South African healthcare worker, including those in KwaZulu-Natal (Reese *et al.*, 2021).

Literature on the impact and experiences of healthcare workers regarding COVID-19 is reasonably well documented globally, reporting on the range of challenges faced by frontline health workers and the multifaceted implications thereof (Bennett *et al.*, 2020; Muhi *et al*., 2020; Peiffer-Smadja *et al.*, 2020; Walton *et al*., 2020). The findings of a qualitative phenomenological study to obtain in-depth understanding of healthcare workers’ experiences regarding the COVID-19 crisis in China revealed that healthcare workers’ experiences centred on a tremendous sense of responsibility towards affected patients. Moreover, participants also reported experiencing fear related to the possibility of being infected and transmitting infection to others (Liu *et al.*, 2020). Another study to explore healthcare worker perceptions and experiences during the COVID-19 pandemic in the United Kingdom reported that limited personal protective equipment (PPE) and lack of routine testing perpetuated experiences of anxiety and distress among health worker, subsequently resulting in a tangible impact on the health workforce (Vindrola-Padros *et al.*, 2020). Similarly, a hybrid inductive-abductive analysis of healthcare workers’ experiences and well-being during the COVID-19 pandemic in the United States reported the experience of health worker distress attributed to the negative impact of poor government and health system leadership, non-compliance of community members with COVID-19-related regulations and the general medical misinformation that spread during the initial outbreak of the disease (Hennein and Lowe, 2020). In another study conducted in Nigeria, experiences of difficulty working in a new environment and challenges of limited resources were reported among healthcare workers at the height of the COVID-19 pandemic (Okediran *et al.*, 2020).

The results of earlier studies on previous pandemics of severe acute respiratory syndrome (SARS) and Middle East Respiratory Syndrome (MERS), also revealed experiences of psychological distress, fear, stigma and high rates of infection among frontline healthcare workers (Robertson *et al.*, 2004; Almutairi *et al.*, 2018; Lee *et al.*, 2018). Similar findings were also reported during the Ebola outbreak, mainly attributed to poor public health preparedness Smith *et al.* (2017); (Belfroid *et al.*, 2018).

In South Africa, particularly KwaZulu-Natal, there is a paucity of data on how COVID-19 has been experienced by healthcare workers, especially those working at the primary healthcare (PHC) level. Policy-related data on health systems reveal that South Africa, including KwaZulu-Natal, has adopted the re-engineered PHC approach to deliver a comprehensive range of health services, with the district health system being the vehicle to drive this approach (Ijumba *et al.*, 2015; Marcus *et al*., 2017). Research on the delivery of healthcare at PHC level has recorded several challenges experienced by healthcare workers such as poor resources allocation, staffing challenges contributing to higher workload, longer patient-wait times, increasing client complaints and generally lower levels of job satisfaction (Kautzky and Tollman, 2008; Marais and Petersen, 2015; Maphumulo and Bhengu, 2019). The reported challenges are noted to be more prominent in low-income and rural primary care settings (Suri *et al*., 2007; Mburu and George, 2017). The present COVID-19 incidence rates coupled with related morbidity are expected to exacerbate these burdens at the PHC level, even in KwaZulu-Natal. To that end, the purpose of the present study was to qualitatively explore PHC practitioners’ experiences regarding the COVID-19 pandemic in KwaZulu-Natal, South Africa. The results have potential implications for health policy and practice, particularly pertaining to psychosocial support, education and training of PHC practitioners. This study emanates in part from a larger study to explore PHC practitioner reflections regarding the COVID-19 pandemic in KwaZulu-Natal, South Africa.

## Methods

### Study design

We employed a qualitative approach using a descriptive cross-sectional design to explore and describe PHC practitioners’ experiences regarding the Coronavirus (COVID-19 pandemic) in KwaZulu-Natal, South Africa.

### Setting

The study was conducted at two different PHC facilities, namely a community health centre and satellite clinic in a purposively selected health district in the KwaZulu-Natal province of South Africa. The health facilities selected form part of the public health system, which belongs to the Department of Health. The selected health facilities are both located in an area which is rural, serving an under-resourced, underdeveloped and mostly unemployed community. The community health centre is located in the northern part of the KwaZulu-Natal province and serves a catchment population of 65 000 people in a rural community. The average headcount of the facility is 22 000 people per month who use the health facility to access an array of comprehensive PHC services which include chronic disease management (TB, HIV and non-communicable diseases), treatment of minor ailments and provision of maternal, women and child health service, the facility also provides mobile health service in hard to reach areas within the catchment community. Services in the community health centre are provided by various members of the multi-disciplinary health team which includes doctors, nurses, physiotherapist, occupational therapist, clinical associates, radiographers, social workers and community caregivers. The selected satellite clinic is also located in the northern part of the KwaZulu-Natal province and serves a catchment population of 25 000 people living in the community. The average number of people who use the facility is 7000 every month. This facility provides also provides comprehensive PHC services using a one-stop shop approach with service delivery being primarily nurse-led.

The clinics selected for data collection were in the geographical location that was nearest to the senior author, thus allowing for an in-depth and immersive understanding of the participants’ experiences within the context of the dynamics of the respective communities.

### Sampling and recruitment strategies

Purposive sampling was used to achieve the desired result. Purposive sampling is especially useful for investigating unusual situations and participants are chosen for a specific reason which is peculiar to them (Leedy and Ormrod, 2001:219; Neuman, 2006:222). Hence, 35 healthcare workers were viewed as key informants as they are frontiers in this pandemic. For the study, PHC practitioners included workers who were permanently employed in the two selected clinics for a duration of at least six months, while volunteers, students, temporarily employees and those who were working for a duration of less than six months at the PHC were excluded. The sample size constituted of nurses, clinical associates, pharmacists, social workers and community caregivers. However, medical doctors, dentists and occupational therapists elected not to participate. A clash in schedules was the main hindrance from collecting data. A reschedule was proposed but this was turned down for unknown reasons. Hence, the researcher did not pursue further because any forced interactions would taint the quality of data. As a result, sample scope became limited to nurses, clinical associates, pharmacists, social workers and community caregivers. The age of participants ranged from 20 to 55 years (See Table 1 for demographic details of participants).

**Table 1.** Profile of participants

Participant	Unique Code	Gender	Professional Category	Duration working as a healthcare professional	Duration working at a PHC facility	Duration working at current PHC facility	Type of exposure to COVID-19
One	P01	Male	Clinical Nurse Practitioner	8 years	4 years	4 years	Suspected and confirmed cases
Two	P02	Female	Social worker	10 years	4 years	4 years	Suspected cases
Three	P03	Male	Physiotherapist	8 Months	8 Months	8 Months	Suspected cases
Four	P04	Female	Operational Nursing Manager	10 Years	5 Years	3 Years	Suspected and confirmed cases
Five	P05	Male	Nutritionist	7 years	4 Years	4 Years	None
Six	P06	Female	Pharmacy Manager	13 Years	5 Years	5 Years	None
Seven	P07	Male	Professional Nurse	3 Years	2 Years	2 Years	Suspected and confirmed cases
Eight	P08	Male	Clinical Associate	3 Years	3 Years	3 Years	Suspected and confirmed cases
Nine	P09	Female	Community Caregiver	4 Years	4 Years	2 Years	Suspected and confirmed cases
Ten	P10	Male	Enrolled Nurse	5 Years	5 Years	5 Years	Suspected and confirmed cases
Eleven	P11	Female	Enrolled Nursing Auxiliary	8 Years	7 Years	7 Years	Suspected and confirmed cases
Twelve	P12	Female	Professional Nurse	6 Years	3 Years	3 Years	Suspected and confirmed cases
Thirteen	P13	Male	Clinical associate	1 Year	1 Year	1 Year	Suspected and confirmed cases
Fourteen	P14	Female	Community Caregiver	2 Years	2 Years	2 Years	Suspected and confirmed cases
Fifteen	P15	Male	Professional Nurse	18 Months	18 Months	18 Months	Suspected and confirmed cases

Initial identification and recruitment of study participants was done through mediated access, which involved obtaining permission from participants’ immediate supervisors so as to obtain buy-in for carrying out the study. Supervisors were assisted by providing information on participants that would be relevant to the study based on the study’s inclusion criteria. Participants were then approached by the researcher. All participants provided informed consent to participate in this study. A total number of 35 participants were targeted to participate in the study. However, a total number of 15 interviews were conducted because of the point of content saturation. The researcher observed that there were no new themes that were emerging; hence, to avoid repetition it was decided to stop at 15 interviews.

### Data collection

Individual, one-on-one in-depth interviews using a semi-structured interview guide were used to collect data. The data collection instrument comprised of two sections – the first was related to demographic details of the participants and the second was related to semi-structured interview guide developed by the senior author with questions related to participants’ experiences of COVID-19 in KwaZulu-Natal, South Africa. Data were collected between April 2020 and September 2020. All interviews were conducted in English, and an audiotape was used to record the interview. The duration of each interview ranged from 20 to 55 min. Thirty-five participants were targeted; however, 25 participants were willing to be part of the study.

All interviews were completed at the end of the day after the participants had completed their clinical duties. To maintain privacy, the interviews were conducted in a private consulting room in both clinics, and COVID-19 protocols were followed. Due to the COVID-19 government regulations in effect at the time, all COVID-19 safety precautions were observed by use of PPE (such as surgical mask) by participants and the senior author during the interviews. Surfaces were also sanitised before and after each interview in each of the consulting rooms where interviews were conducted. Social distancing was maintained, and hand hygiene practices were followed before and after each interview.

A pre-test was done with one healthcare worker prior commencement of the actual study to validate the instrument. No changes were made to the original data collection instrument based on the initial data collected.

### Data analysis

Data were analysed concurrently with data collection. Recruitment of participants ceased after data content saturation was reached. Content data saturation was reached at the 15^th^ participant. The collected data were transcribed verbatim and then analysed thematically using content analysis. Vaismoradi *et al*. (2013:399) explain that thematic analysis aims to qualify and analyse narrative data on social life. Narratives describe how people live their daily lives, practices and subjective perceptions, which can be communicated orally or in written texts (Neuman, 2006:474). Thus, thematic analysis was key to data analysis. Tesch’s (2013) method of data analysis for qualitative research was followed. Data were analysed in collaboration with experts in qualitative research methodology to ensure trustworthiness. All the authors also reviewed the codebook, categories and the themes that emerged from the data. Disagreements were discussed, and consensus was reached after further deliberations. The analysis of data was an iterative process, which entailed continuous reading and re-reading of the interview transcripts. The transcripts were consistently reviewed and compared with audio-recorded data to ensure the reliability and credibility of the research findings.

### Ethical considerations

Ethical approval to conduct this study was obtained from the Biomedical Research Ethics Committee of the University of KwaZulu-Natal (BREC/00001446/2020). Approval to conduct the study was also obtained from the KwaZulu-Natal Department of Health (NHRD Ref: KZ_202007_015). Informed consent was obtained verbally and in writing prior to data collection from all participants. Participants were also given a written information sheet to ensure they understood the nature of the study.

## Results

In the section that follows, we identified three broad themes surrounding participants’ experiences related to the COVID-19 pandemic in two PHC settings in KwaZulu-Natal. These themes are as follows: (1) personal experiences, (2) occupational experiences and (3) community and patient-related experiences. In addition, there were 12 superordinate themes, 6 of which included 15 subordinate themes while 6 had none. The superordinate themes were psychological distress, self-stigma, disruption of social norm, epiphany, conflict of interest, staff infections, COVID-19-related courtesy stigma, resource constraints, poor dissemination of information and struggles with societal issues, clinician-patient relations and COVID-19-related mismanagement of patients. The themes surrounding these experiences are discussed with the associated subthemes and exemplar quotes, with a summary provided in Table 2.

**Table 2.** Summary of themes and subthemes

1. Personal experiences	
1.1. Psychological distress	Anxiety and depression Emotional trauma Pseudo-symptoms
1.2. Self-stigma	Self-blame Self-isolation and withdrawal
1.3. Disruption of social norm	Reality of lockdown Adjusting to new normal
1.4. Epiphany	Reality of COVID-19 pandemic
1.5. Conflict of interest	

2. Occupational experiences	
2.1. Staff infections	
2.2. COVID-19-Related Courtesy stigma	Isolation Blame Labelling and stereotyping
2.3. Resource constraints	Poor supply of PPE Poor-quality PPE Unfavourable physical infrastructure Shortage of staff and increased absenteeism
2.4. Poor dissemination of information	

3. Community and patient-related experiences	
3.1. Struggles with societal issues 3.2. Nurse-patient relations 3.3. COVID-19-related mismanagement of patients	

### Personal experiences

The first theme described the experiences of participants related to COVID-19 in KZN, South Africa, concerned personal experiences which yielded five superordinate subthemes, namely psychological distress, self-stigma, disruption of social norm, epiphany and conflict of interest.

#### Psychological distress

Three subordinate themes related to the personal experience of psychological distress emerged from the data. These are (a) anxiety and depression, (b) emotional trauma and (c) pseudo-symptoms.

##### Anxiety and depression

Participants in this study described physiological symptoms such as headaches, feelings of uncertainty and that they were on the edge. This experience highlighted the sense of anxiety and depression that was felt by the participants. The following excerpts support this theme:

‘there are a lot of uncertainties because we are seeing people getting tested and we are seeing people awaiting their results getting agitated. They are anxious. Some are depressed….’ (Participant2, Female, Social worker)

‘You feel like you are getting a headache, you are getting this and that. I think that it is just anxiety, more than anything else. It has been anxiety for me’. (Participant5, Male, Nutritionist)

##### Emotional trauma

Participants in this study reported feeling emotions that were akin to being emotionally overwhelmed and having the sensation of being hurt emotionally to such an extent that some lacked the motivation to be able to work. This was supported by the following statements:

‘That is the issue because even as health practitioners, you come to work, it is like you are drained by just being at work because you are scared for your life’. (Participant7, Male Professional Nurse)

‘I so wish that this COVID-19 can go away because it is traumatising us’. (Participant4, Female, operational nursing manager)

##### Pseudo-Symptoms

This subordinate theme emerged from the data in which participants reported the COVID-19-like symptoms that they would experience particularly when in the working environment. Typically, these symptoms would occur mainly when they received news of colleagues or close acquaintances being infected with COVID-19.

‘Some people will feel that chest pain even if it is not really there…. Not that it is fake, but psychologically’. (Participant1, Male, Clinical nurse practitioner)

‘Just knowing or hearing that some close to you has caught COVID-19 makes you start to feel sick, I remember when I first heard that one of our colleagues had it, like almost immediately I started to feel like my throat was sore and had a headache …I though even I was sick at that moment’. (Participant2, Female, Social worker)

#### Self-stigma

Two subordinate themes related to the experience of self-stigma emerged from the data. These are (a) self-blame and (b) self-isolation.

##### Self-blame

Participants reported how they felt personally responsible for others being infected with COVID-19 in the workplace. These participants were mainly those that were infected with COVID-19, and they also expressed how they also blamed themselves for not taking adequate precautions to protect themselves and their families.

‘In my department I was the first one to get infected then after that the rest of the staff went to test, some were positive and others were negative…. I feel guilty because I got it first and I think I infected others’. (Participant7, Male, Professional nurse)

‘I don’t know maybe I could have done more to protect myself and family, I feel like it’s my fault that people at home got exposed to the disease because of the work that I do’. (Participant11, Female, Enrolled nursing auxiliary)

##### Self-Isolation

The participants who had a history of COVID-19 symptoms and previous infection reported that they often avoided social gathering in the community and interaction with colleagues in the work place due fear of knowing what other perceived about them.

‘When I had the symptoms, I tested and still had to work but I stayed away from everyone else because I was afraid that everyone would think that I have COVID-19 and will infect them so even in the morning I would avoid morning meetings and gatherings at lunch’. (Participant7, Male, Professional Nurse)

‘I just stayed indoors during my quarantine period and even after I did not visit anyone because I was scared of what people will think or say now that I had this disease’. (Participant 12, Female, Professional Nurse)

#### Disruption of the social norm

The experience of disruption of the social norm yielded two subordinate themes, namely (a) reality of lockdown and (b) disruption of status quo.

##### Reality of lockdown rules

Participants reported experiences pertaining to regulations that were instilled to enforce lockdown as a measure of curbing the spread of COVID-19. Participants reported fears regarding non-adherence to the rules and how they had to quickly adapt to new changes as a result of these regulations.

‘Even worse, you are afraid to go to the funeral because of the restriction of 50 people or less, plus you do not know how many people at the funeral had interacted with them before they discovered they are sick’. (Participant4, Female, Operational nursing manager)

‘Everything just changed all of a sudden, we had to adjust to so many rules all at the same time, even going out to restaurants was not allowed, we had to be in doors at a specific time, number of people going in shops was reduced so even shopping was a bit of a hassle because of Queues’. (Participant8, Male, Clinical associate)

##### Disruption of the status quo

Participants reported how the pandemic resulted in changes in the working environment such as the enforcing of social distancing within the health facilities and mandatory wearing of masks. Participants also reported how changes brought about by COVID-19 impacted on clinical services in terms of service delivery.

‘Services like rehabilitation have been distracted by lockdown regulations…. There’s also been the new norm of social distancing etc, I have to now constantly tell patients in the queue that they must wear their masks properly and make sure that they nose and mouth is covered’ (Participant3, Male, Physiotherapist)

‘It became so hard because you have to do all the clinical programmes by yourself because you are alone due to everyone being sick of COVID’. (Participant4, Female, operational nurse manager)

#### Epiphany

Epiphany as a superordinate theme only yielded one subordinate theme namely reality of the COVID-19 pandemic.

##### Reality of the COVID-19 pandemic

Participants reported having to come to terms with the fact that COVID-19 was a reality due to the number of people that were testing positive in the workplace. This was supported by the following statements:

‘I think because now it has hit the, well it has hit home, type of thing, because most people are testing positive and more staff are testing positive’ (Participant 2, Female, Social worker)

‘it is becoming a reality that COVID-19 is here to stay…. that affects you as a person, especially as a healthcare worker, if you see your fellow healthcare workers being posted on social media and people sending their condolences. You are in the same line of duty’ (Participant7, Male, Professional Nurse).

#### Conflict of interest

The superordinate theme of conflict of interest was a standalone theme which highlighted participants’ internal debate between values of the health professional and their personal well-being as ordinary individuals. The following statements support this superordinate theme:

‘All these circulars are telling us about things that are impossible to achieve…. I think we should be following the guidelines, whatever guidelines that are there, but I am not seeing them being practical in the situation that we are currently in right now’. (Participant3, Male, Physiotherapist)

‘You cannot be compromising your life at the expense of a patient. You cannot be wanting or focusing or wanting to achieve the optimum health of your client if you are sick. So, there is a dichotomy there between you being exposed to COVID-19 and wanting to prevent your patient from contracting COVID-19’. (Participant1, Male, Clinical nurse practitioner)

### Occupational experiences

The second category of description in experiences of participants related to COVID-19 in KZN, South Africa, concerned occupational experiences which yielded four superordinate themes, namely staff infections, courtesy stigma, resource constraints and poor dissemination of information. The four superordinate themes subsequently yielded seven subordinate themes.

#### Staff infections

The superordinate theme of staff infections emerged from the data as a standalone theme which revealed participants’ description of how COVID-19 infected healthcare workers, participants describe what they believed were the contributing factors for healthcare workers being infected with COVID-19. This was supported by the following statements:

‘Nurses were infected by the patients; some patients were not aware that they were infected with COVID. They just come to you with other problems and not mention that they are also infected because they are also not sure’. (Participant7, Male, Professional nurse)

‘So, during the time of eating, your mask is off and you are preparing to eat. There was not social distancing of 1.5m. So, it is where the problem started because while you are eating, they are also talking, so they infected one another’. (Participant2, Male, Enrolled nurse)

#### COVID-19-related courtesy stigma

The superordinate theme of COVID-19-related courtesy stigma emerged from the data and describes the stigma that participants experienced as a result of working in direct contact with a suspected confirmed COVID-19-infected patient. From the data, courtesy stigma was perpetrated by other health professionals who had not been in contact with a COVID-19 patient. In this regard, participants who were in contact with COVID-19 patients branded a mark of shame, fear and isolation. Three subordinate themes associated with courtesy stigma emerged from the data, these are (a) isolation, (b) blame and (c) labelling and stereotyping.

##### Isolation

Participants reported stigma in how certain colleagues would not want to associate or socialise with a staff member who had been infected with COVID-19. Participants also expressed how they were socially excluded from certain interactions such as unit meetings. This was supported by the following statements:

‘Even the ones that are working with that person are stigmatising that person, it is like they are also feeling like why is this one at work and not at home, because you have tested positive yet you are coming to work’. (Participant2, Female, Social worker)

‘During the morning meetings I would often see everyone is groups talking and keeping far from me even the way they would look at me or react when I would arrive in the room, you could just see that they want me to stay far from them. This occurred for weeks even after I returned from isolation’. (Participant7, Male, Professional nurse)

##### Blame

Participants reported how they were assigned responsibility for contributing to staff-related infections that occurred from COVID-19, participants reported feeling and experiencing bad treatment from fellow co-workers to sometimes expressed words of blame related to staff infection stemming from COVID-19. The following statements support this subordinate theme:

‘When they test positive they feel like they are being treated badly, as if they brought the virus to the clinic’. (Participant2, Female, Social worker)

‘I was one of the first people to get infected in my unit and my experience was not good because my colleagues would be weary around me, at other times I would even hear them saying that COVID-19 came with me in the unit’. (Participant7, Male, Professional nurse)

##### Labelling and stereotyping

This subordinate theme emerged from the data in which participants reported how certain co-workers would make remarks that were indicative of a general misperception regarding the spread of COVID-19. These remarks would also result in increased feelings of isolation and an erection of social barriers between healthcare workers.

‘Obviously, we are going to be infected by the fact that you are here with us, you know’. (Participant12, Female, Professional nurse)

‘There was this colleague I worked with who wanted nothing to do with COVID-19 even touching a patients file who was a suspect was a problem with her. She would say things like “you are carrying a COVID-19 file …you are going to give us COVID with the file you are carrying, go away from here with that file.”’ (Participant 15, Male, Professional Nurse)

#### Resource constraints

The superordinate theme of resource constraints highlighted the challenges associated with all types of resources that were necessary for working effectively during the COVID-19 pandemic. Four subordinate themes associated with this superordinate theme emerged from the data. The emerging subordinate themes were as follows: (a) poor supply of PPE, (b) poor quality of PPE, (c) inconducive working environment and (d) shortage of staff (increasing absenteeism).

##### Poor supply of PPE

Participants reported that there were supplied with inadequate PPE; in other cases, there was none at all, and this was a challenge to them. This was supported by the following statements:

‘There are people who are expected to trace and test without PPE, are receiving COVID patients without PPE. Now, PPE has been an issue since before COVID’. (Participant10, Male, Enrolled nurse)

‘We did not have enough PPE to cover ourselves during this pandemic. So, that was the main challenge…. there was nothing at all’. (Participant 5, Male, Nutritionist)

##### Poor quality of PPE

Participants reported that in cases where PPE was supplied, there were challenges with the quality of the various PPE supplied. Issues related to quality of PPE related to surgical masks and hand sanitizers. This was supported by the following statements:

‘Even the PPE that we were using like masks, it was not surgical masks. It was paper masks, which were very poor’. (Participant13, Male, Clinical associate)

‘The hand sanitizer formulation, you find three different formulations in one batch. You find that this bottle is clear, this bottle is like jelly, this bottle is runny, you know, from one batch. This is supposed to be one batch with the same consistency and same quality, but you find that there are three different formulations in one. That is one. Two, when it comes to masks, I have heard nurses complaining that you find that you wear a surgical mask, then it tears off’. (Participant6, Female, Pharmacy manager)

##### Unfavourable physical infrastructure

Participants reported that their working environments were not suitable to ensure that reduction of the spread of COVID-19 infection. Alluding, participants revealed how the health facility infrastructure were not able to allow for social distancing even in the staff rooms and thus possibly contributed to the staff-related infections that occurred. Participants also revealed how the working conditions contributed to increasing infection rates as staff with suspected COVID-19 infection were tested and asked to remain at work. The following statements support this subordinate theme:

‘with us in this clinic I have heard that people were exposed positive colleagues and were told to test and continue working…. Some ended up positive and did not even isolate or quarantine because results arrived so late and they were working while waiting for these results’ (Participant8, Male, Clinical associate)

‘A lot of staff related infections occurred because the tea rooms were not conducive to social distancing and as a result may workers infected each other because they had lunch without masks obviously and were in close proximity with one another’. (Participant4, Female, Operational nurse manager)

##### Shortage of staff and increasing absenteeism

Participants reported challenges with availability of sufficient staff due to increasing number of healthcare workers that became infected with COVID-19. Participants reported how the shortage of staff had a negative impact on the quality of health service delivered as fewer staff were available to meet the demand of various patients and health programmes at PHC level.

‘Sometimes you would find that you are alone, working for 10 nurses who are away because some are isolated because they have tested positive. Some are in quarantine. So, it is only you and a few others. So, there was a lot of work that I experienced. There was a lot of work and patients were coming in large numbers’. (Participant15, Male, Professional nurse)

‘The issue of staffing was a big challenge particularly in the beginning when we did not have dedicated COVID staff as the infections in staff were increasing and so was the workload. I think in that regard; service delivery was severely affected but we tried to do our best with the little that we had’. (Participant6, Female, Pharmacy manager)

#### Poor dissemination of information

The superordinate theme of poor dissemination of information had no subordinate themes. This theme emerged from the data in which participants reported challenges with how information was communicated in terms of accuracy, frequency and clarity. Typically, participants reported that there were delays in information sharing from higher levels within the health system. Moreover, information shared was often not clear nor could it be applicable as its contents were not contextualised to individual health settings. This was supported by the following statements:

‘when it comes to disseminating the information to the healthcare workers, there was not much, even from the Department. When information came, it was very late we had already been doing what we were thinking was right only to find that it’s not. Also, the information provided came from national most of the time and was always changing especially in the beginning and that caused a lot of confusion on its own’ (Participant6, Female, Pharmacy manager)

‘I believe that the institution or the institution’s management does not disseminate information the way they should. We sometimes have to dig for information ourselves and ask them what to do in certain situations. They do not freely give certain information’. (Participant13, Male, Clinical associate)

### Community and patient-related experiences

The third category of description in experiences of participants related to COVID-19 in KZN, South Africa, concerned community and patient-related experiences which yielded three superordinate themes, namely (a) struggles with societal issues, (b) nurse-patient relations and (c) mismanagement of patients. The results of this section are discussed only in relation to these three superordinate themes as there were no emerging subordinate themes.

#### Struggles with societal issues

Participants reported the challenges they faced at community level regarding adherence to behavioural interventions to curb the spread of COVID-19. Participants particularly expressed concern regarding the public transport system which was unable to adhere to stipulated regulations, which they believe put their lives at risk. In this regard, participants seemed to have a degree of helplessness and hopelessness as the situation was seemingly beyond their control. The following excerpts support this superordinate theme:

‘I use public transport. Some people just cough. You cannot demand that the windows in the taxi be opened at all times while the taxi is travelling a long distance. Is the taxi yours? It is cold in the morning. people are cold, and then you demand that they open windows’. (Participant9, Female, Community caregiver)

‘Getting to work during the lockdown period was particularly stressful because there was fewer taxis operating and the times of working were reduced …getting to work on time was a challenge over and above the fact that some did not adhere to the COVID-19 regulations that were set at the time’ (Participant7, Male, Professional nurse)

#### Clinician-patient relations

Participants described the relation that they had with patients to be generally positive in that patients understood the situation and changes brought about by COVID-19. Moreover, participants stated that patients seemed to have a trust and belief in them during this pandemic period, evidence by a dependency on them for information related to COVID-19 among other things. This was supported by the following statements:

‘Our patients have been very understanding. I will say so, because we have never really had a very hectic period or uprising from the patients’ side. I think they have been very understanding because even when it comes to screening, there has not been a time where people felt that if you are standing in front of me you will infect me’. (Participant6, Female, Pharmacy manager)

‘Overall I think patients have tried to adapt to new ways of working however there are some that still don’t want to wear masks properly but when you call them out for it they listen. In this period, I have seen most of them really been depending of us for information on this disease among man thing’. (Participant14, Female, Community caregiver)

#### COVID-19-related Mismanagement of patients

Certain participants revealed that patients were not being managed correctly in terms of the stipulated screening and isolation procedure that were set. Pertaining to clinical care of suspected patients, there was also a general mismanagement in terms of medical care. This was supported by the following statements.

‘Patient tells the screener that they are coughing, the screener sometimes just decides that the patient is fine and they tick “no” in the screening form. Where does that patient end up? They end up coming to you’. (Participant8, Male, Clinical associate)

‘In the early stages of the pandemic there were lots of challenges with treatment of suspected COVID-19 cases, you would find doctors ordering antibiotics for patients without confirming whether that patient has COVID or not.…Steroids were being ordered when they should not be ordered and even isolation principles were not be observed fully when care was being provided’. (Participant6, Female, Pharmacy manager)

## Discussion

Given the lack of literature on healthcare practitioners’ experiences during COVID-19 pandemic, the present sought to add to the literature on the experiences of healthcare working in KwaZulu-Natal, South Africa, during the COVID-19 pandemic. The findings yielded three broad themes of description in participants’ experiences of the phenomenon, namely (1) personal experiences, (2) occupational experiences and (3) community and patient-related experiences. Five superordinate subthemes emerged from participants’ personal experiences related to the COVID-19 pandemic, namely psychological distress, self-stigma, disruption of the social norm, epiphany and conflict of interest. Four superordinate subthemes emerged from participants’ occupational experiences related to the COVID-19 pandemic in KZN, South Africa. These are as follows: staff infections, courtesy stigma, resource constraints and poor dissemination of information. Three superordinate themes emerged from the community and patient-related experiences; these were as follows: struggles with societal issues, clinician-patient relations and mismanagement of patients.

In this study, participants reported psychological distress in the form of anxiety and depression, emotional trauma and pseudo-symptoms as a personal experience of the COVID-19 pandemic in KwaZulu-Natal, South Africa. Al-Hanawi *et al.* (2020) also reported high levels of COVID-19-related psychological distress among healthcare workers in Saudi Arabia. Similar findings were also cited in other parts of the world where psychological distress was mainly attributed to the presence of COVID-19 symptoms and fear of possibly being infected due to direct patient contact (Gómez-Salgado *et al.*, 2020; Shacham *et al.*, 2020; Li *et al.*, 2021).

The personal experience of self-stigma was also reported in this study and yielded subordinate subthemes of self-blame and self-isolation. Self-stigma is an internalised negative stereotype that is associated with an individual’s illness (Maharjan and Panthee, 2019). Self-stigma is often associated with low self-esteem, low self-efficacy and poor quality of life (Dinesh Mittal *et al.*, 2012). According to Mahmoudi *et al.* (2021), self-stigma is an important factor related to mental distress during the COVID-19 pandemic and necessitates interventions to improve mental health among individuals affected by COVID-19. The reported findings of COVID-19 self-stigma among healthcare workers in this study are a significant finding as COVID-19 self-stigma has mainly been cited in studies examining patients affected by COVID-19 (Aleid *et al.*, 2020; Grover *et al.*, 2021; Mahmoudi *et al.*, 2021). While a similar study to explore COVID-19-related stigma and its associated factors among Egyptian physicians revealed the prevalence of self-stigma among healthcare workers, the manifestation of such stigma and contributing factors was mainly related to fear of exposing one’s family to COVID-19 infection (Mostafa *et al*., 2020).

Disruption of the social norm was also an experience reported by participants and was attributed to the reality of lockdown rules and disruption of the status quo, which were subordinate subthemes emerging from this reported experience. Globally, the COVID-19 pandemic has resulted in the disruption of lives and livelihoods in several areas of life. A study on the impact of lockdown on the experiences and practices of breast feeding among new mothers in the United Kingdom revealed that lockdown has negatively impacted women resulting in distress and other challenges with healthcare during the antenatal and postnatal period (Vazquez-Vazquez *et al.*, 2021). Another study on the impact of COVID-19 restrictions on individual living with dementia in England reported negative psychological and cognitive effects due to the imposed restrictions (Tuijt *et al.*, 2021). In Ghana, it was found that COVID-19 restrictions adversely affected individuals economically due to the disruptions in routine business operations (Adom *et al*., 2020).

Coming to terms with the reality of the COVID-19 pandemic was another personal experience reported by participants. Alluding, participants revealed how the increasing rates of infection among peers validated the authenticity of the pandemic. Since the beginning of the pandemic, healthcare workers have increasingly experienced high levels of exposure to COVID-19, which has subsequently resulted in a several healthcare workers being infected with or dying from the virus (Alajmi *et al.*, 2020; Stock *et al.*, 2020; Sabetian *et al.*, 2021). A recent review of literature on COVID-19 infection rates among healthcare workers has revealed a total of 152, 888 healthcare workers having been infected with COVID-19 worldwide (Bandyopadhyay *et al.*, 2020). South Africa has experienced an unprecedented number of healthcare workers being infected with COVID-19, while there have been just over 1300 COVID-19-related case fatalities among healthcare workers. The KwaZulu-Natal province is among the top three leading regions with the highest COVID-19 morbidity and mortality among healthcare workers healthcare (Nxumalo and Mchunu, 2021).

The personal experience of epiphany was also related to the reality of the lockdown regulations that were imposed by government in an attempt to curb the spread of COVID-19. Participants reported challenges with adjusting and adhering to these regulations. This finding represents a potential threat to public health as the COVID-19 pandemic is an unprecedented global health challenge which has been proven to be reliant on strict adherence to lockdown measures in order to curb the spread of the disease (Coetzee and Kagee, 2020). The findings from a study to understand the patterns of adherence to COVID-19 mitigation measures revealed that the individual decision to adhere to regulations was rooted in an individuals’ perceived susceptibility to exposure to the virus, thus highlighting the need for a more nuanced understanding of the adherence to lockdown measures and the intrinsic motivations of the individual (Denford *et al.*, 2021).

Participant’s personal experiences of the COVID-19 pandemic also revealed a conflict of interest wherein values of the health profession were in question with regard to their personal safety. This finding represents one of the many ethical dilemmas that maybe faced by healthcare providers during course of providing care to clients which may affect healthcare workers willingness to perform the duties required of them. The findings of A recent study to explore ethical dilemmas and healthcare workers willingness to work amid the COVID-19 pandemic suggested that certain Palestinian healthcare workers were unwilling to work amid the COVID-19 pandemic (Maraqa *et al*., 2021). Similarly, the findings of early studies on previous outbreaks such as SARS revealed that healthcare workers faced major ethical dilemmas during these outbreaks which were mostly related to conflicting reports, truth telling and resource allocation (Bernstein and Hawryluck, 2003). The present study findings are a confirmation of existing conflicting values faced by healthcare workers when caring for COVID-19 patients, which may require the provision of a supportive climate that nurtures critical reflection regarding ethical issues and the provision of tailored support strategies to address related concerns (Sperling, 2020).

Staff infections was an occupational-related experience reported by participants, which revealed contributing factors such as lack of in-facility social distancing that was believed to have resulted in staff infections. This finding is consistent with other research studies that have revealed high rates of risk and infection rates among healthcare workers as opposed to non-healthcare providers (Ali *et al.*, 2020a; Zheng *et al.*, 2020; Wong *et al.*, 2021). Collectively, these findings highlight the need for ensuring consistent availability of necessary resources to minimise healthcare workers risk of being infected with COVID-19.

Courtesy stigma was also an experience reported in this study and manifested in the form of isolation, blame and labelling, and stereotyping. Courtesy stigma, which has also previously been referred to as stigma by association or associative stigma, refers to a mark of shame, fear or negative connotation that an individual may be subjected to by virtue of their association with someone of something that maybe be deemed as deviant by a minority or majority (Östman and Kjellin, 2002). While research on COVID-19-related stigma has been reasonably documented (Duan *et al*., 2020; Villa *et al.*, 2020; Yuan *et al.*, 2021), there remains a dearth of research on courtesy stigma related to COVID-19, especially as it pertains to healthcare workers. The present literature available of courtesy stigma of COVID-19 related to health workers has revealed that it may be associated with high levels of psychological distress, which may in turn have a negative impact of job satisfaction and service delivery (Zandifar *et al.*, 2020).

Participants in this study reported experiences of resources constraints in the form of poor quantity and quality of PPE, unfavourable physical infrastructure and increasing absenteeism with subsequent shortages in staffing. Ahmed *et al.* (2020) reported that there was a general lack of different forms of PPE in the United States and Pakistan. A review of the contributing factors to the shortage of PPE during the COVID-19 pandemic revealed that this was a global challenge, especially during the first wave of the pandemic and was mainly attributed to dysfunctional costing models adopted by health facilities and an unprecedented high demand for supply which depleted PPE inventories (Cohen and Rodgers, 2020). The present study findings of poor quality of PPE supplied rarely been cited in research related to PPE challenges faced during COVID-19 and may be attributed to the demand and supply issues that have been mentioned in previous studies (Jessop *et al.*, 2020; Shrivastava and Shrivastava, 2020). Similar to safety concerns reported due to the shortage of PPE, the presence of poor-quality PPE also has the potential to exacerbate existing anxiety related to health workers perceived safely and susceptibility to infection thus resulting and poor mental health outcomes which has the potential to subsequently hinder the provision of optimum quality of health services. Participant’s experience of an unfavourable physical infrastructure is a significant finding as it is reflective of the possible contributing factors to the high numbers of COVID-19-related infections that have been noted among frontline healthcare workers. Moreover, such findings are indicative of a lack of resilience in the health system and organisational response to the pandemic. According to Lloyd-Smith (2020) the resilience of healthcare systems in the present novel circumstances surrounding the COVID-19 pandemic are reliant on the rapid construction of a new order of systems and processes by making creative use of available resources an structure and enabling their recombination. The experience of increasing absenteeism with accompanying shortages of staff was also another experience reported by participants. Increasing absenteeism can also be attributed to staff infections and subsequent resultant shortages of staff. The challenge of staffing shortages is not unique to South Africa, but is a global phenomenon that has been reported widely in healthcare settings around the world (Anderson and Ruhs, 2010; Monsalud *et al.*, 2021). As the pandemic progress, there are major concerns around the added stress on the workforce, leading to further shortages.

In South Africa, challenges in staffing are prominent in the rural context and have been reported at primary care level (Kautzky and Tollman, 2008; Moosa *et al*., 2017) The existing rates of both communicable and non-communicable diseases that are prevalent in South Africa have contributed burn out and increasing levels of poor job satisfaction among healthcare workers, particularly nurses (Delobelle *et al.*, 2011). The present incidence, prevalence and related COVID-19 mortality and morbidity in South Africa adds to the present challenges faced by healthcare workers during delivery of health services, especially in KwaZulu-Natal, South Africa. Collectively, the superordinate themes of staff infections, COVID-19-related stigma and resource constraints highlight the dynamics of changing workload, changing roles and changing burden of disease brought on by the COVID-19 pandemic in KwaZulu-Natal, South Africa. Alluding, Jensen and McKerrow (2021) as well as Pillay *et al.* (2021) have described the impact of the COVID-19 pandemic on PHC services in South Africa, highlighting issues of disruptions in service access and delivery due to staff limitations and complications related to rising COVID-19 infections.

Poor dissemination of information within the Department of Health, especially from higher levels within the health system, were also reported in this study. Challenges with communication were related to the accuracy, frequency and clarity in the manner of disseminating information pertaining to COVID-19, associated control measures and appropriate clinical and non-clinical management thereof. This finding is significant as it suggests that healthcare workers may not be fully equipped to render the appropriate healthcare due to the resultant misinformation that may stem from poor dissemination of information. According to Garrett (2020), the present COVID-19 pandemic has also been complicated by a deluge of misinformation about the nature of the disease, which impeded effective management strategies to curb and managed the pandemic. Healthcare workers especially at primary care level play a pivotal role in the outbreak response in terms of preventive and curative strategies to control the pandemic. The availability of sufficient, accurate and succinct information is thus imperative if healthcare workers are to institute effective interventions for patients (Tran *et al.*, 2020).

Community-related experiences in the form of struggles with community issues, clinician-patient relations and mismanagement of patients were also reported in this study. Struggles with societal issues were an experience reflecting healthcare workers challenges with community members regarding adherence to behavioural interventions to curb the spread of COVID-19. This finding is consistent with those of Smith *et al.* (2020) which have revealed poor compliance of community member with COVID-19 lockdown regulations. On the other hand, the findings of another similar study revealed that high level of compliance with government regulations instituted to curb the spread of COVID-19 in Vietnam (Nguyen *et al.*, 2020). Literature on the factors associated with adherence and non-adherence to COVID-19 control measures has revealed that compliance and non-compliance were associated with a variety of individual perceptual and contextual factors among these being the behaviours of others, perceived susceptibility to COVID-19 infection and perspectives about the severity of infection (Bellato, 2020; Hills and Eraso, 2021; Reinders Folmer *et al.*, 2021).

Clinician-patient relations as a finding highlights the level of importance that patients place on the health workers and the health system. This finding represents an important entry point for which behaviour modification interventions can be founded especially as it pertains to preventing the spread of COVID-19 at an individual and community level.

Mismanagement of patients was also reported in this study and was mainly related to clinical care in terms of COVID-19 screening and management of patients under investigation. This finding represents a general lack of adequate preparation of healthcare workers which may be rooted in the poor dissemination of information which was reported by participants in this study.

## Conclusion

The present study findings reveal those PHC practitioners’ experiences regarding the COVID-19 pandemic stem from the multitude of psychosocial consequences that have arisen as a result of the global catastrophe caused by the disease. The reported experiences maybe associated with poor mental health outcomes, decreased job satisfaction and the potential for poor health service delivery. The findings of this study suggest that poor pandemic preparedness on the part of government authorities within the ministry of health is central to the challenges reported. Ensuring the constant availability of reliable and contextually relevant sources of information could be valuable in helping healthcare workers cope with living and working during the pandemic.
